# Crystal structure of a second monoclinic polymorph of 3-meth­oxy­benzoic acid with *Z*′ = 1

**DOI:** 10.1107/S2056989018016900

**Published:** 2019-01-01

**Authors:** Tze Shyang Chia, Huey Chong Kwong, Qin Ai Wong, Ching Kheng Quah, Md. Azharul Arafath

**Affiliations:** a X-ray Crystallography Unit, School of Physics, Universiti Sains Malaysia, 11800 USM, Penang, Malaysia; b School of Chemical Sciences, Universiti Sains Malaysia, 11800 USM, Penang, Malaysia; c Department of Chemistry, Shahjalal University of Science and Technology, Sylhet, 3114, Bangladesh

**Keywords:** crystal structure, polymorph, hydrogen-bond, 3-meth­oxy­benzoic acid

## Abstract

A new polymorphic form of the title compound, C_8_H_8_O_3_, is described in the centrosymmetric monoclinic space group *P*2_1_/*c* with *Z*′ = 1 as compared to the first polymorph, which crystallizes with two conformers (*Z*′ = 2) in the asymmetric unit in the same space group. In the crystal of the second polymorph, inversion dimers linked by O—H⋯O hydrogen bonds occur and these are linked into zigzag chains, propagating along the *b*-axis direction by C—H⋯O links. The crystal structure also features a weak π–π inter­action, with a centroid-to-centroid distance of 3.8018 (6) Å. The second polymorph of the title compound is less stable than the reported first polymorph, as indicated by its smaller calculated lattice energy.

## Abstract 2

A second polymorph of 3-meth­oxy­benzoic acid with *Z*′ = 1 is reported and compared with first polymorph with *Z*′ = 2.

## Chemical context   

Meth­oxy­benzoic acid, also called anisic acid, consists of three isomers with mol­ecular formula C_8_H_8_O_3_: the crystal structures of 2- and 4-meth­oxy­benzoic acids with *Z*′ = 1 have been reported (Parvez, 1987 ▸; Etter *et al.*, 1988 ▸; Bryan, 1967 ▸; Colapietro & Domenicano, 1978 ▸; Fausto *et al.*, 1997 ▸; Hathwar *et al.*, 2011 ▸) and polymorphism has not been observed for these two isomers in the Cambridge Structural Database (CSD) (Version 5.39, last update August 2018; Groom *et al.*, 2016 ▸) to date. In this article, we report a second polymorphic form (Iβ) of 3-meth­oxy­benzoic acid with *Z*′ = 1 and compare its properties with those of the previously reported first polymorphic form (Iα). Polymorph Iα crystallizes in the monoclinic space group *P*2_1_/*n* with *a* = 13.8034 (17) Å, *b* = 5.0275 (5) Å, *c* = 21.446 (3) Å and β = 99.320 (13)° (Raffo *et al.*, 2014 ▸; refcode EFINEO). The asymmetric unit of Iα consists of two mol­ecules with different conformations (*Z*′ = 2), which are connected into a homodimer through strong O—H⋯O hydrogen bonds. As described below, these two conformers (*A* and *B*) differ in the orientation of the meth­oxy group and its relative position from the —OH group. DFT calculations suggest that the *A* conformer of Iα is more energetically stable than the *B* conformer (Pereira Silva *et al.*, 2015 ▸).
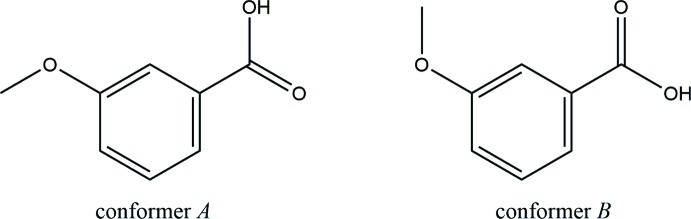


## Structural commentary   

**Figure 1:**
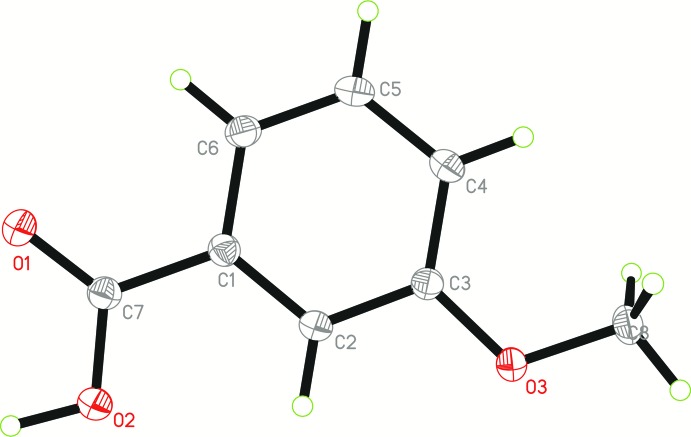
The mol­ecular structure of Iβ with 50% probability displacement ellipsoids.

The asymmetric unit of Iβ (Fig. 1 ▸) consists of a unique 3-meth­oxy­benzoic acid mol­ecule (*Z*′ = 1). The mol­ecule is almost planar with a maximum deviation of 0.107 (1) Å at atom O1. The mol­ecules of Iβ adopt a similar conformation (overlay r.m.s.d. = 0.052 Å) as compared to the conformer *A* of Iα (Raffo *et al.*, 2014 ▸). The carboxyl group (O1/O2/C7/H1*O*2) of Iβ is close to coplanar with the attached phenyl ring (C1–C6) as indicated by the dihedral angle of 5.6 (7)°. The C8—O3—C3—C2 torsion angle of Iβ is −176.63 (7)° as compared to −176.75 (11) and −1.4 (2)° for conformers *A* and *B*, respectively, of Iα.

## Supra­molecular features   

**Figure 2:**
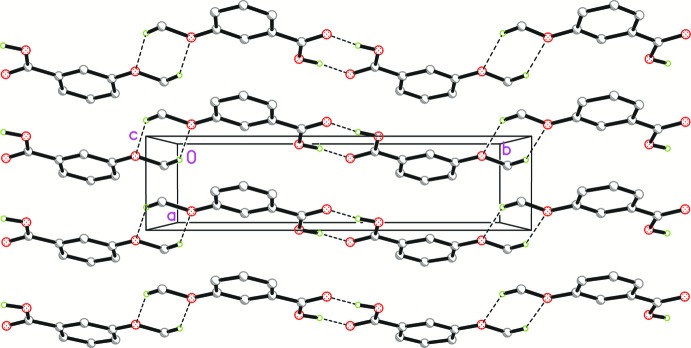
Partial crystal packing of Iβ. Dashed lines represent the hydrogen-bonds. Hydrogen atoms not involved in hydrogen bonding are omitted for clarity.

In the crystal of Iβ, two inversion-related mol­ecules are joined into a homodimer with an 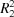
(8) graph-set motif via strong pairwise O—H⋯O hydrogen bonds (Fig. 2 ▸, Table 1 ▸). The homodimers are linked by weak C—H⋯O hydrogen bonds between two meth­oxy groups into zigzag chains with 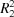
(6) graph-set motifs, which propagate along the *b*-axis direction. The [010] chains are stacked along the *a* axis into corrugated sheets parallel to the *ab* plane via weak π–π inter­actions with a centroid-to-centroid distance of 3.8018 (6) Å (symmetry codes: *x* − 1, *y*, *z* and *x* + 1, *y*, *z*) and slippage of 1.676 Å.

## Hirshfeld surface analysis   

**Figure 3:**
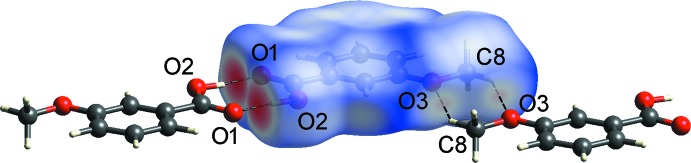
The Hirshfeld surface mapped over *d*_norm_ of the central mol­ecule of Iβ hydrogen bonded to two neighbouring mol­ecules.

**Figure 4:**
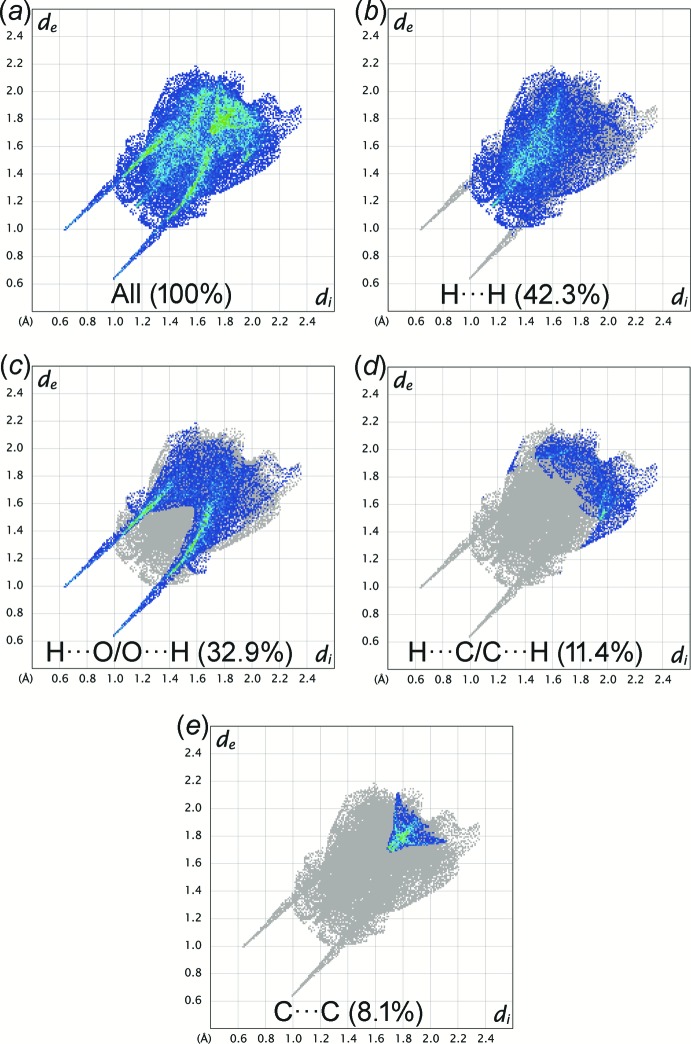
The two-dimensional fingerprint plots of Iβ for different inter­molecular contacts giving their percentages of contribution to the Hirshfeld surface. *d*_i_ and *d*_e_ are the distances from the Hirshfeld surface to the nearest atom inter­ior and exterior, respectively, to the surface.

The Hirshfeld surfaces mapped with normalized contact distance *d*_norm_ and the two-dimensional fingerprint plots for Iβ were generated using *CrystalExplorer17.5* (Turner *et al.*, 2017 ▸). The large and small red spots on the Hirshfeld surface mapped with *d*_norm_ (Fig. 3 ▸) correspond to the O2—H1*O*2⋯O1 and C8—H8*A*⋯O3 hydrogen bonds, respectively. The H⋯O distances are 1.09 and 0.16 Å shorter than the sum of van der Waals radii of H and O atoms (2.72 Å). The H⋯H contact is the most populated contact and contributes 42.3% of the total inter­molecular contacts, followed by H⋯O/O⋯H (32.9%), H⋯C/C⋯H (11.4%) and C⋯C (8.1%) contacts (Fig. 4 ▸). The tips of pseudo-mirrored sharp spikes at *d*_e_ + *d*_i_ ≃ 1.6 Å represent the shortest H⋯O/O⋯H contacts, corresponding to the O2—H1*O*2⋯O1 hydrogen-bond. The absence of significant C—H⋯π inter­action in the crystal structure of Iβ is indicated by the absence of characteristic ‘wings’ in the fingerprint plot of H⋯C/C⋯H contacts. The C⋯C contacts include the weak π–π inter­action, which appears as a unique ‘triangle’ focused at *d*_e_ ≃ *d*_i_ ≃ 1.8 Å. The π–π inter­action is illustrated as a unique pattern of red and blue ‘triangles’ on the shape-index surface and a flat region on the curvedness surface of the phenyl ring (see supporting Figures S1 and S2).

## Lattice energy calculation   

The lattice energies of polymorphs Iα and Iβ were calculated using *PIXEL* software (Gavezzotti, 2003 ▸) at default settings. The calculated lattice energy of Iα (107.5 kJ mol^−1^) is larger than that of Iβ (98.5 kJ mol^−1^) and this comparison is in agreement with the report of Pereira Silva *et al.* (2015 ▸), in which Iα is more stable than Iβ under ambient conditions.

## Database survey   

For the structure of 2-meth­oxy­benzoic acid (refcodes FUFBOX and FUFBOX01, respectively), see: Parvez (1987 ▸) and Etter *et al.* (1988 ▸). For the structure of 4-meth­oxy­benzoic acid (refcodes ANISIC, ANISIC01, ANISIC02 and ANISIC04, respectively), see: Bryan (1967 ▸), Colapietro & Domenicano (1978 ▸), Fausto *et al.* (1997 ▸) and Hathwar *et al.* (2011 ▸). For the previous structure of 3-meth­oxy­benzoic acid (refcodes EFINEO and EFINEO01, respectively), see: Raffo *et al.* (2014 ▸) and Pereira Silva *et al.* (2015 ▸).

## Synthesis and crystallization   

Single crystals of Iβ were obtained from an unsuccessful attempt of co-crystallization between 3-meth­oxy­benzoic acid and hexa­methyl­ene­tetra­mine. Colourless plate-like crystals were obtained from slow evaporation of a methano­lic mixture of 3-meth­oxy­benzoic acid and hexa­methyl­ene­tetra­mine in equimolar ratio at room temperature.

## Refinement   

Crystal data, data collection and structure refinement details are summarized in Table 2 ▸. The O-bound H atom was located from the difference-Fourier map and refined freely [O2—H1*O*2 = 1.01 (2) Å]. The remaining H atoms were positioned geometrically [C—H = 0.95 and 0.98 Å] and refined using a riding model with *U*_iso_(H) = 1.2 or 1.5*U*_eq_(C). A rotating group model (AFIX 137) was applied to the methyl group.

## Supplementary Material

Crystal structure: contains datablock(s) I. DOI: 10.1107/S2056989018016900/hb7789sup1.cif

Structure factors: contains datablock(s) I. DOI: 10.1107/S2056989018016900/hb7789Isup2.hkl

Hirshfeld shape-index and curvedness Figures. DOI: 10.1107/S2056989018016900/hb7789sup3.docx

Supporting information file. DOI: 10.1107/S2056989018016900/hb7789Isup4.cml

CCDC reference: 1448794

Additional supporting information:  crystallographic information; 3D view; checkCIF report

## Figures and Tables

**Table 1:** Hydrogen-bond geometry (Å, °)

*D*—H⋯*A*	*D*—H	H⋯*A*	*D*⋯*A*	*D*—H⋯*A*
O2—H1*O*2⋯O1^i^	1.008 (19)	1.626 (19)	2.6295 (9)	173.3 (17)
C8—H8*A*⋯O3^ii^	0.98	2.56	3.4016 (11)	144

Symmetry codes: (i) 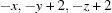; (ii) 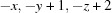.

**Table 2:** Experimental details

Crystal data	
Chemical formula	C_8_H_8_O_3_
*M*_r_	152.14
Crystal system, space group	Monoclinic, *P*2_1_/*c*
Temperature (K)	100
*a*, *b*, *c* (Å)	3.8018 (4), 15.6027 (16), 11.9755 (12)
β (°)	90.889 (2)
*V* (Å^3^)	710.28 (13)
*Z*	4
Radiation type	Mo *K*α
μ (mm^−1^)	0.11
Crystal size (mm)	0.56 × 0.22 × 0.12

Data collection	
Diffractometer	Bruker SMART APEXII DUO CCD
Absorption correction	Multi-scan (*SADABS*; Bruker, 2009 ▸)
*T*_min_, *T*_max_	0.881, 0.987
No. of measured, independent and observed [*I* > 2σ(*I*)] reflections	9395, 2550, 2049
*R*_int_	0.023
(sin θ/λ)_max_ (Å^−1^)	0.758

Refinement	
*R*[*F* ^2^ > 2σ(*F* ^2^)], *wR*(*F* ^2^), *S*	0.038, 0.113, 1.04
No. of reflections	2550
No. of parameters	105
H-atom treatment	H atoms treated by a mixture of independent and constrained refinement
Δρ_max_, Δρ_min_ (e Å^−3^)	0.40, −0.27

Computer programs: *APEX2* and *SAINT* (Bruker, 2009 ▸), *SHELXS2013* and *SHELXTL* (Sheldrick, 2008 ▸), *SHELXL2014* (Sheldrick, 2015 ▸), *PLATON* (Spek, 2009 ▸) and *publCIF* (Westrip, 2010 ▸).

## supplementary crystallographic information

### Crystal data

**Table:**

C_8_H_8_O_3_	*F*(000) = 320
*M**_r_* = 152.14	*D*_x_ = 1.423 Mg m^−^^3^
Monoclinic, *P*2_1_/*c*	Mo *K*α radiation, λ = 0.71073 Å
*a* = 3.8018 (4) Å	Cell parameters from 3889 reflections
*b* = 15.6027 (16) Å	θ = 2.6–32.4°
*c* = 11.9755 (12) Å	µ = 0.11 mm^−^^1^
β = 90.889 (2)°	*T* = 100 K
*V* = 710.28 (13) Å^3^	Plate, colourless
*Z* = 4	0.56 × 0.22 × 0.12 mm

### Data collection

**Table:**

Bruker SMART APEXII DUO CCD diffractometer	2550 independent reflections
Radiation source: fine-focus sealed tube	2049 reflections with *I* > 2σ(*I*)
Graphite monochromator	*R*_int_ = 0.023
φ and ω scans	θ_max_ = 32.6°, θ_min_ = 2.1°
Absorption correction: multi-scan (*SADABS*; Bruker, 2009)	*h* = −5→5
*T*_min_ = 0.881, *T*_max_ = 0.987	*k* = −21→23
9395 measured reflections	*l* = −18→17

### Refinement

**Table:**

Refinement on *F*^2^	Primary atom site location: structure-invariant direct methods
Least-squares matrix: full	Hydrogen site location: mixed
*R*[*F*^2^ > 2σ(*F*^2^)] = 0.038	H atoms treated by a mixture of independent and constrained refinement
*wR*(*F*^2^) = 0.113	*w* = 1/[σ^2^(*F*_o_^2^) + (0.0616*P*)^2^ + 0.1229*P*] where *P* = (*F*_o_^2^ + 2*F*_c_^2^)/3
*S* = 1.04	(Δ/σ)_max_ = 0.001
2550 reflections	Δρ_max_ = 0.40 e Å^−^^3^
105 parameters	Δρ_min_ = −0.27 e Å^−^^3^
0 restraints	

### Special details

**Table:**

Geometry. All esds (except the esd in the dihedral angle between two l.s. planes) are estimated using the full covariance matrix. The cell esds are taken into account individually in the estimation of esds in distances, angles and torsion angles; correlations between esds in cell parameters are only used when they are defined by crystal symmetry. An approximate (isotropic) treatment of cell esds is used for estimating esds involving l.s. planes.

### Fractional atomic coordinates and isotropic or equivalent isotropic displacement parameters (Å^2^)

**Table:**

	*x*	*y*	*z*	*U*_iso_*/*U*_eq_	
O1	0.20200 (18)	0.96657 (4)	0.88546 (5)	0.02247 (16)	
O2	−0.04494 (18)	0.88699 (4)	1.01876 (5)	0.02142 (16)	
H1O2	−0.091 (5)	0.9451 (12)	1.0523 (16)	0.068 (5)*	
O3	0.17686 (17)	0.58676 (4)	0.92170 (5)	0.01959 (15)	
C1	0.2204 (2)	0.81461 (6)	0.86862 (7)	0.01512 (16)	
C2	0.1573 (2)	0.73618 (6)	0.91952 (7)	0.01515 (16)	
H2A	0.0504	0.7341	0.9906	0.018*	
C3	0.2516 (2)	0.66069 (5)	0.86565 (7)	0.01517 (16)	
C4	0.4082 (2)	0.66360 (6)	0.76109 (7)	0.01698 (17)	
H4A	0.4738	0.6121	0.7245	0.020*	
C5	0.4674 (2)	0.74261 (6)	0.71099 (7)	0.01847 (18)	
H5A	0.5727	0.7447	0.6396	0.022*	
C6	0.3755 (2)	0.81821 (6)	0.76329 (7)	0.01759 (17)	
H6A	0.4169	0.8718	0.7283	0.021*	
C7	0.1244 (2)	0.89573 (6)	0.92491 (7)	0.01636 (17)	
C8	0.2877 (2)	0.50822 (6)	0.87173 (7)	0.01985 (18)	
H8A	0.2229	0.4601	0.9198	0.030*	
H8B	0.5434	0.5091	0.8628	0.030*	
H8C	0.1726	0.5017	0.7984	0.030*	

### Atomic displacement parameters (Å^2^)

**Table:**

	*U*^11^	*U*^22^	*U*^33^	*U*^12^	*U*^13^	*U*^23^
O1	0.0301 (3)	0.0151 (3)	0.0223 (3)	−0.0001 (2)	0.0063 (2)	0.0011 (2)
O2	0.0285 (3)	0.0176 (3)	0.0184 (3)	0.0009 (2)	0.0077 (2)	−0.0004 (2)
O3	0.0254 (3)	0.0134 (3)	0.0202 (3)	0.0012 (2)	0.0073 (2)	0.0000 (2)
C1	0.0147 (3)	0.0151 (4)	0.0156 (3)	0.0003 (3)	0.0006 (2)	−0.0005 (3)
C2	0.0151 (3)	0.0165 (4)	0.0139 (3)	0.0002 (3)	0.0018 (2)	−0.0004 (3)
C3	0.0146 (3)	0.0150 (4)	0.0159 (3)	0.0000 (3)	0.0013 (2)	0.0001 (3)
C4	0.0161 (3)	0.0190 (4)	0.0160 (3)	0.0008 (3)	0.0025 (3)	−0.0026 (3)
C5	0.0175 (4)	0.0231 (4)	0.0149 (4)	−0.0005 (3)	0.0035 (3)	−0.0001 (3)
C6	0.0182 (4)	0.0183 (4)	0.0164 (4)	−0.0009 (3)	0.0023 (3)	0.0021 (3)
C7	0.0164 (3)	0.0170 (4)	0.0157 (3)	0.0006 (3)	0.0008 (3)	0.0001 (3)
C8	0.0217 (4)	0.0141 (4)	0.0238 (4)	0.0017 (3)	0.0033 (3)	−0.0029 (3)

### Geometric parameters (Å, º)

**Table:**

O1—C7	1.2394 (10)	C3—C4	1.3956 (12)
O2—C7	1.3110 (10)	C4—C5	1.3909 (12)
O2—H1O2	1.01 (2)	C4—H4A	0.9500
O3—C3	1.3666 (10)	C5—C6	1.3829 (12)
O3—C8	1.4301 (10)	C5—H5A	0.9500
C1—C2	1.3896 (12)	C6—H6A	0.9500
C1—C6	1.4019 (11)	C8—H8A	0.9800
C1—C7	1.4823 (12)	C8—H8B	0.9800
C2—C3	1.3925 (12)	C8—H8C	0.9800
C2—H2A	0.9500		
			
C7—O2—H1O2	109.8 (11)	C6—C5—H5A	119.4
C3—O3—C8	116.94 (7)	C4—C5—H5A	119.4
C2—C1—C6	120.52 (8)	C5—C6—C1	119.09 (8)
C2—C1—C7	120.47 (7)	C5—C6—H6A	120.5
C6—C1—C7	119.01 (8)	C1—C6—H6A	120.5
C1—C2—C3	119.62 (7)	O1—C7—O2	122.85 (8)
C1—C2—H2A	120.2	O1—C7—C1	121.76 (7)
C3—C2—H2A	120.2	O2—C7—C1	115.39 (7)
O3—C3—C2	115.43 (7)	O3—C8—H8A	109.5
O3—C3—C4	124.26 (7)	O3—C8—H8B	109.5
C2—C3—C4	120.31 (8)	H8A—C8—H8B	109.5
C5—C4—C3	119.34 (8)	O3—C8—H8C	109.5
C5—C4—H4A	120.3	H8A—C8—H8C	109.5
C3—C4—H4A	120.3	H8B—C8—H8C	109.5
C6—C5—C4	121.13 (8)		

### Hydrogen-bond geometry (Å, º)

**Table:**

*D*—H···*A*	*D*—H	H···*A*	*D*···*A*	*D*—H···*A*
O2—H1*O*2···O1^i^	1.008 (19)	1.626 (19)	2.6295 (9)	173.3 (17)
C8—H8*A*···O3^ii^	0.98	2.56	3.4016 (11)	144

Symmetry codes: (i) −*x*, −*y*+2, −*z*+2; (ii) −*x*, −*y*+1, −*z*+2.
