# Supplementary material for: Crystal structure of a second monoclinic polymorph of 3-meth­oxy­benzoic acid with Z′ = 1
Source: Acta Crystallogr E Crystallogr Commun. 2019 Jan 1;75(Pt 1):8–11. doi: 10.1107/S2056989018016900 (PMC6323879; doi:10.1107/S2056989018016900)
Supplement: Supplementary file 3 [file e-75-00008-sup3.docx]

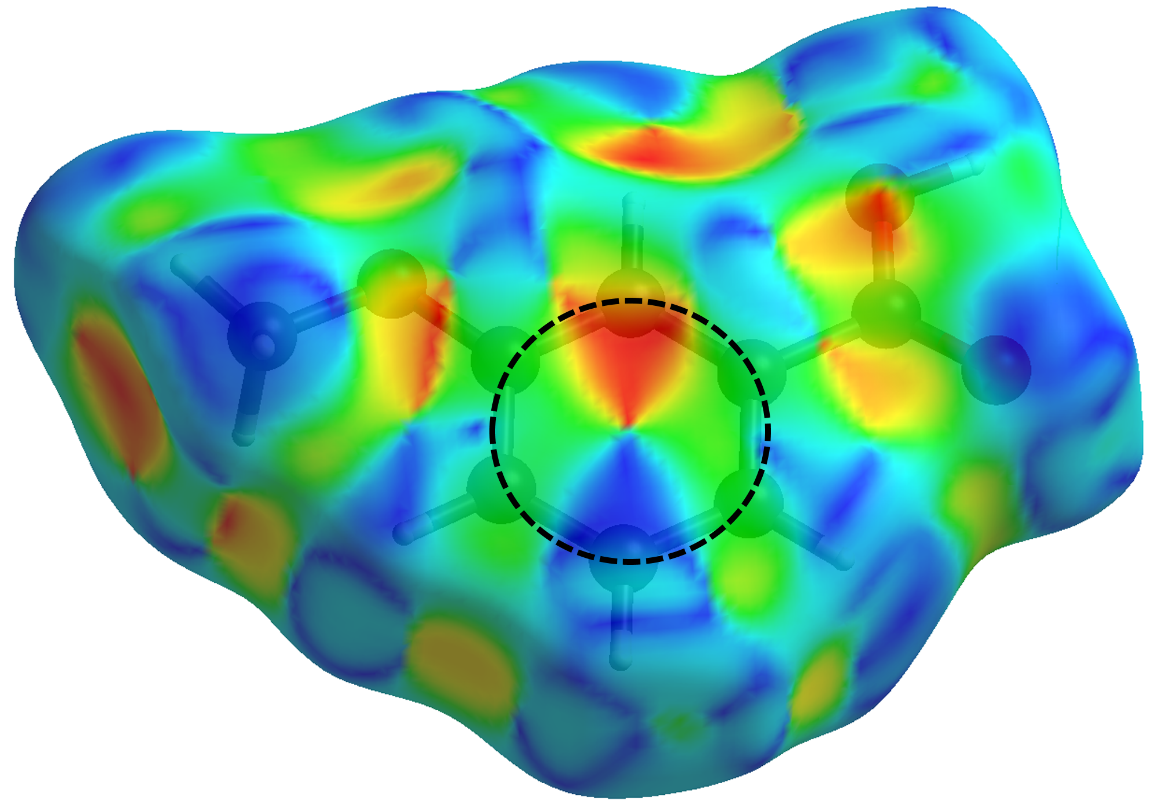


Figure S1: the Hirshfeld surface mapped with shape index of Iβ


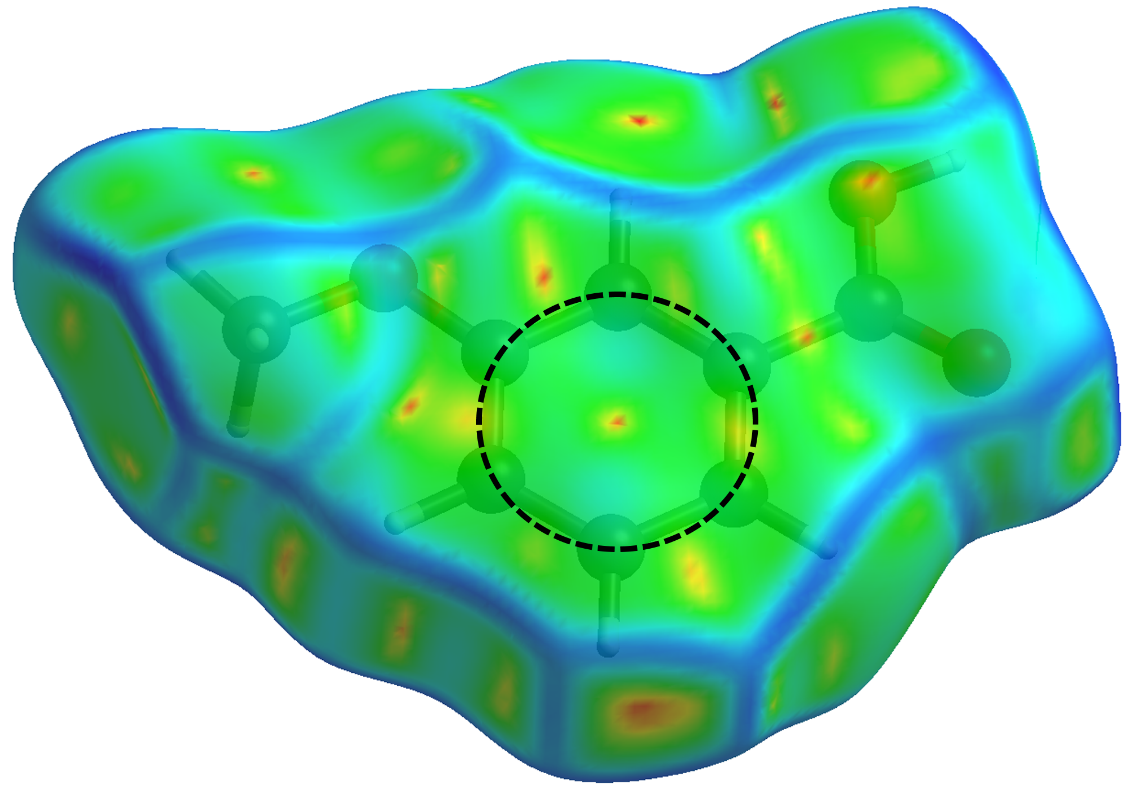


Figure S2: the Hirshfeld surface mapped with curvedness of Iβ
